# Event and Comment

**Published:** 1921-09

**Authors:** 


### DENTAL REGISTER

THE MAGAZINE OF DENTISTRY

Vol. LXXV SEPTEMBER, 1921 No. 9

### EVENT AND COMMENT

**Proposed Plan for the Dental Education of the Public**

We are giving in the editorial pages of this issue an article by W. Linford Smith, which sets forth a scheme that will make it practicable to inform the lay mind as to the essential means by which every one can be intelligent as the proper care of the teeth and the oral conditions of dental health.

The matter has been under consideration for several years by Mr. Smith, and he has now presented it in a form that he believes it a practicable solution and a scheme for increasing dental health of the masses, who give little attention to the importance of such matter. His plan of increasing dental health will largely depend upon what cooperation and help the dental profession is willing to give to his scheme. The matter has been presented to the authorities of the National Dental Association, and at the last meeting of this body it was authorized, and suitable committees were appointed to put the scheme into operation at an early date.

Of course there will be objections to this, as there always are to any new ideas, but since the matter seems to be, on its face at least, wholly organized for the public good, and no one so far has questioned its propriety, we can see no reason why it may not be adopted. At least we can do what is frequently a questionable and dangerous experiment, start it out with the best safe guides we know; and in case we find it will not function desirably we can throw out the switch and stop the machine. Or we can make such repairs as seem to be needed to give the desired results. Mr. Smith and those who believe in his proposition are willing to assume the larger share of the financial burdens involved to at least get the scheme started and to functioning to the point where those most concerned will be willing to assume such reasonable expense as experience will demand or require for some such function as a public necessity.

As the plan seems to indicate a purely altruistic motive, and since it commends itself as being both practicable and ideal, we hope it may meet with commendable consideration and approval, if it seems to be the proper action to take at this time.

We herewith present the entire article and ask a careful consideration before any further action is finally taken.

